# Loss of Homogentisate 1,2-Dioxygenase Activity in *Bacillus anthracis* Results in Accumulation of Protective Pigment

**DOI:** 10.1371/journal.pone.0128967

**Published:** 2015-06-05

**Authors:** Hesong Han, Liudmyla Iakovenko, Adam C. Wilson

**Affiliations:** Department of Biology, Georgia State University, Atlanta, GA, United States of America; University Medical Center Utrecht, NETHERLANDS

## Abstract

Melanin production is important to the pathogenicity and survival of some bacterial pathogens. In *Bacillus anthracis*, loss of *hmgA*, encoding homogentisate 1,2-dioxygenase, results in accumulation of a melanin-like pigment called pyomelanin. Pyomelanin is produced in the mutant as a byproduct of disrupted catabolism of L-tyrosine and L-phenylalanine. Accumulation of pyomelanin protects *B*. *anthracis* cells from UV damage but not from oxidative damage. Neither loss of *hmgA* nor accumulation of pyomelanin alter virulence gene expression, sporulation or germination. This is the first investigation of homogentisate 1,2-dioxygenase activity in the Gram-positive bacteria, and these results provide insight into a conserved aspect of bacterial physiology.

## Introduction

*Bacillus anthracis* is a Gram-positive, endospore-forming bacterium that is the etiological agent of anthrax. Virulence in the mammalian host depends on production of anthrax toxin and capsule, carried on virulence plasmids pXO1 and pXO2, respectively [1]. Expression of both the toxin and capsule genes is regulated by the master virulence regulator AtxA. The activity of AtxA and the production of virulence factors are tied through several regulatory networks to the physiological state of the cell [2–6].

Production of melanin pigments contributes to microbial pathogenesis, as it is associated with virulence in many microorganisms (reviewed in [7]). Melanin can protect cells from oxidative damage, alter phagocytosis and phagocytotic killing, modify virulence factor activity, and change responses to antimicrobial compounds [7–9]. Melanin production can also alter susceptibility to UV damage, protecting microorganisms from environmental damage [10].

Pyomelanin is a melanin-like pigment produced by the spontaneous oxidation of homogentisic acid [11]. Homogentisic acid is an intermediate in the pathway catabolizing L-tyrosine and L-phenylalanine to acetoacetate and fumarate in many species [12–14]. Homogentisic acid normally does not accumulate in significant amounts as it is converted to maleylacetoacetate by the activity of homogentisate 1,2-dioxygenase. In the absence of homogentisate 1,2-dioxygenase, homogentisic acid accumulates in the cell and spontaneously oxidizes to pyomelanin, a water-soluble brown pigment. Pyomelanin is also known as alkaptomelanin in the human genetic disorder alkaptonuria, in which L-tyrosine catabolism is disrupted by the loss of human homogentisate 1,2-dioxygenase, resulting in the accumulation of damaging brown pigment in tissues [14].

In this report, we have identified and characterized the gene encoding homogentisate 1,2-dioxygenase (*hmgA*) of *B*. *anthracis*. Loss of *hmgA* results in accumulation of pyomelanin in the presence of L-tyrosine and L-phenylalanine. Pyomelanin protects *B*. *anthracis* cells from UV damage but loss of *hmgA* does not alter other metabolic and virulence characteristics.

## Materials and Methods

### Bacterial strains and growth conditions

*B*. *anthracis* strain 34F2 (pXO1^+^ pXO2^-^) and its derivatives were routinely grown in LB or brain heart infusion (BHI) broth supplemented with the appropriate antibiotics at the following concentrations: chloramphenicol (7.5μg/ml), spectinomycin (100μg/ml), and kanamycin (7.5μg/ml). As indicated, *B*. *anthracis* was grown in a modified formulation of defined R-Media [15] buffered to pH 7.0 without 0.8% NaHCO_3_ under 5% CO_2_. 5-bromo-4-chloro-3-indolyl-β-D-galactopyranoside (X-Gal) (40μg/ml) was added to LB agar to monitor β-galactosidase activity as necessary. Competent cells of *B*. *anthracis* were prepared following the method of Koehler *et al* [16], and electroporation was performed using the Bio-Rad-Gene Pulser according to the instructions of the supplier.

*E*. *coli* TG1, C600 and DH5α competent cells were used for the propagation and isolation of all plasmid constructs. *E*. *coli* transformation was performed in chemically competent cells as previously described [17]. Transformants were selected on LB agar supplemented with ampicillin (100μg/ml), chloramphenicol (7.5μg/ml), spectinomycin (100μg/ml), or kanamycin (30μg/ml).

### Transposon mutagenesis and analysis

Transposition was performed in the 34F2 strain using the Mariner-based transposon delivery system pAW068 [18] and the protocols previously described [19]. The mutants were screened for altered morphology when grown on LB-agar plates containing spectinomycin.

To identify the site of insertions, mutant strains were grown overnight in BHI containing 0.5% glycerol and spectinomycin at 28°C. Genomic DNA was extracted from the overnight cultures using UltraClean Microbial DNA Isolation Kit (MoBio, Carlsbad, CA). The site of insertion was identified by restriction digestion of genomic DNA using NsiI. Digested genomic DNA was then re-ligated and used to transform *E*. *coli*. The presence of the pUC origin of replication allows re-ligated DNA containing the transposed sequence to replicate in *E*. *coli* as a spectinomycin-resistant plasmid. Following isolation of plasmid from *E*. *coli*, sequencing of transposon-flanking DNA was performed using transposon-specific primers oMarSeq1 (5’-GCTTGTCATCGTCATCCTTGT) and oMarSeq2 (5’-GGCCGCGAAGTTCCTATT).

### Plasmid and strain construction

Strains and plasmids are listed in Table 1. A 723 bp region upstream of BAS0228 was PCR amplified from 34F2 genomic DNA using primers BAS0228U5’Bam (5’-CACCGGATCC GCGTTTGAGCAGCATGTAAA) and BAS0228U3’Sal (5’-TGCGTCGACGGAATTGTACATGTCGTTTATG) while a 638 bp region downstream of BAS0228 was amplified using primers BAS0228D5’Sal (5’-CACCGTCGACAAAGAGGCATGCTCAATAACG) and BAS0228D3’Pst (5’- TGCCTGCAGCGTTACATCCGCAACCATC) using OneTaq DNA polymerase (NEB). The upstream PCR fragment was cloned into the BamHI and SalI restriction sites of pORI-I-SceI [20] to generate plasmid pAW406. The downstream region was then cloned into the SalI and PstI sites of pAW406 to generate pAW407. A 590 bp region upstream of BAS0227 was PCR amplified using primers BAS0227U5’Bam (5’-CACCGTCGACGAAAAGGAAGCAGGTGATGAG) and BAS0227U3’Sal (5’-TGCGTCGACTCATAGGGAGGAATCATGATGA) while a 610 bp region downstream of BAS0227 was amplified using primers BAS0227D5’Sal (5’-CACCGTCGACGAAAAGGAAGCAGGTGATGAG) and BAS0227D3’Pst (5’-TGCCTGCAGCTCACAAAACGGGCTATGCT) using OneTaq DNA polymerase. The upstream PCR fragment was cloned into the BamHI and SalI restriction sites of pORI-Cm-I-SceI to generate plasmid pAW416. The downstream region was then cloned into the SalI and PstI sites of pAW416 to generate pAW417. A region containing the coding region of BAS0028 was amplified using primers BAS0228U5’Bam and BAS0228U3’Sal. The PCR product was digested with SnaBI and BamHI and blunt-ended with T4 DNA polymerase. The PCR product was then cloned into pAW285, digested with EcoRI and blunt-ended with T4 DNA polymerase, to generate complementation plasmid pAW444. The sequence of all plasmids was verified by DNA sequencing. Marker-less gene deletions in *B*. *anthracis* were generated through a modification of the technique of Janes and Stibitz [21], as previously described [22].

**Table 1:** Strains and plasmids used in this study.

Strain or plasmid	Relevant Characteristics	Source
***B*. *anthracis* strains**		
34F2	pXO1^+^ pXO2^-^	Laboratory stock
34F2-tM104	Mariner transposon insertion into BAS0028	This study
AW-A127	Markerless deletion of BAS0228 (Δ*hmgA*)	This study
AW-A130	Markerless deletion of BAS0227 (Δ*hmgB*)	This study
**Plasmids**		
pORI-Cm-I-SceI	pORI-Cm vector with I-SceI recognition site, Cm^R^	[20]
pSS4332	I-SceI expression plasmid, Kan^R^	[34]
pTCVlac-*pagA*	*pagA*-*lacZ* transcriptional fusion in pTCV-lac, Kan^R^	[3]
pTCVlac-*atxA12*	*atxA*-*lacZ* transcriptional fusion in pTCV-lac, Kan^R^	[20]
pAW068	Mariner transposon delivery plasmid, Cm^R^ Spc^R^	[18]
pAW285	Xylose-inducible expression plasmid, Cm^R^	[22]
pAW406	Region upstream of BAS0228 in pORI-Cm-I-SceI, Cm^R^	This study
pAW407	Regions flanking BAS0228 in pORI-Cm-I-SceI, Cm^R^	This study
pAW416	Region upstream of BAS0227 in pORI-Cm-I-SceI, Cm^R^	This study
pAW417	Regions flanking BAS0227 in pORI-Cm-I-SceI, Cm^R^	This study
pAW444	BAS0228 coding region in pAW285, Cm^R^	This study

### Vis-NIR Absorption Spectroscopy

Cultures of parental and mutant strains were grown for 48 hours at 37°C. Cell-free supernatants were prepared by centrifugation of cultures at 10,000xg followed by transfer of supernatant to new tubes. The optical spectra of the supernatants were recorded on a Cary 5000 UV-Vis-NIR spectrophotometer at room temperature in 50 mM potassium phosphate buffer pH 7.5 as previously described [23].

### Cell-free extract analysis

Overnight cultures of *B*. *anthracis* strains grown in LB were diluted to OD_600_ of 0.01 in fresh LB and grown for 6 hours at 37°C. Cells were pelleted by centrifugation and resuspended in phosphate-buffered saline. Cells were then disrupted by sonication, cell debris removed by filtration, and cell-free extracts transferred to new tubes. Amino acids were added as indicated to cell-free extracts at the following concentrations based on composition of R-medium: L-phenylalanine 125mg/L; L-tryptophan 35mg/L; L-tyrosine 144mg/L. After overnight incubation at 37°C, samples were assayed visually for color change and spectroscopically at OD_400_.

### UV and H_2_O_2_ sensitivity tests

For UV protection assays, *B*. *anthracis* strains were grown in LB at 37°C for 28 hours. The cultures were transferred into a petri dish and irradiated with UV at 302 nm as indicated in figure. Serial dilutions were performed and plated on LB-agar. After 18 hours at 37°C, colony forming units (CFU) were counted.

For H_2_O_2_ sensitivity by disk diffusion analysis, *B*. *anthracis* strains were grown in LB at 37°C to an OD_600_ of 0.6. Aliquots of bacterial cultures were then added to LB-agar cooled to 50°C and poured into petri dishes. Cooled and solidified plates were overlaid with H_2_O_2_-saturated paper disks at concentrations indicated. Plates were incubated overnight at 37°C and the diameters of the zones of clearance were measured.

H_2_O_2_ protection was also assayed in liquid cultures similar to assays described previously [24]. *B*. *anthracis* strains were grown in LB at 37°C for 24 or 48 hours. Bacteria were then pelleted by centrifugation and resuspended in cell-free supernatants. H_2_O_2_ was added at concentrations indicated and cultures incubated for 15 minutes at 37°C. Serial dilutions were performed and plated on LB-agar. After 18 hours at 37°C, CFU of pre- and post-treatment cultures were counted.

### Sporulation and germination analysis

Sporulation assays were performed using strains grown in SM (Schaeffer’s sporulation medium) [25] at 37°C for 48 hours. Sporulation was initially monitored by visualization of cells on a Zeiss Axio Imager microscope. Sporulation efficiency was assayed by chloroform treatment and enumeration of spores and vegetative cells as previously described for *B*. *anthracis* [22]. Sporulation efficiency is presented as percentage of spores relative to total viable cells.

For germination experiments, strains were grown in SM media for 72 hours at 37°C. Spores were harvested by heating samples to 80°C for 30 minutes followed by 3 washes in sterile water. Spores were enumerated by microscopy using a hemocytometer. 5x10^3^ spores were resuspended in 1 ml LB broth, incubated for 60 minutes at 37°C, serially diluted, and plated on LB-agar. After 18 hours at 37°C, CFU were counted.

### β-Galactosidase assays

*B*. *anthracis* strains harboring gene promoter fusions on the replicative vector pTCV-lac were grown at 37°C in LB supplemented with kanamycin. β-galactosidase activity was assayed as described previously and specific activity was expressed in Miller units [26,27].

## Results

### Transposon mutagenesis generates a pigment-producing mutant

**Fig 1:**
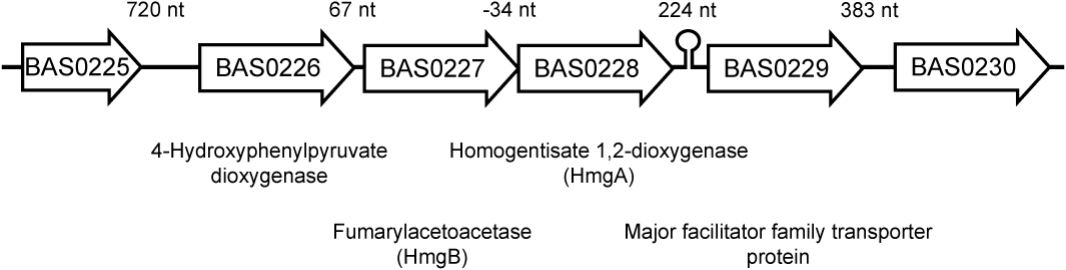
Genetic organization of BAS0228 (*hmgA*) region. Numbers above specify size of intergenic regions. Predicted transcriptional terminator indicated downstream of *hmgA*.

The 34F2 (pXO1^+^ pXO2^-^) strain of *B*. *anthracis* was randomly mutagenized by Mariner transposition using plasmid pAW068. pAW068 carries a Mariner-based transposon system which delivers a transposon containing a spectinomycin-resistance cassette for selection of transposon mutants and a Gram-negative origin of replication for plasmid rescue and identification of mutants [18]. Spectinomycin-resistant insertional mutants were generated as previously described [18,28], plated on LB-agar containing spectinomycin, and incubated for 24 hours at 37°C. Colonies derived from one transposon mutant, annotated 34F2-tM104, became a light brown color after 24 hours. After continued incubation for an additional 24 hours at 37°C, the colonies became dark brown and a brown pigment was observed in the solid media surrounding the mutant colony. Sequencing of the transposon insertion site of the pigmented mutant identified an insertional site 127 nt downstream of the predicted translation start site of BAS0228. BAS0228 is predicted to encode a homogentisate 1,2-dioxygenase. Homogentisate 1,2-dioxygenase, also known as HmgA, has been shown to be involved in the catabolism of L-tyrosine and L-phenylalanine in several species, converting homogentisate to maleylacetoacetate [12–14]. Loss of HmgA activity results in accumulation of homogentisate, which oxidizes to form a brown pigment known as pyomelanin. *hmgA*-like genes are found among members of the *Bacillus cereus* group, such as *B*. *cereus*, *B*. *anthracis*, and *B*. *thuringiensis*, but are not found in the related model organism *Bacillus subtilis* or in other pathogenic firmicutes. Genetic organization of the BAS0228 region is shown in Fig 1. There is a 34 nt overlap between the 5’ end of *hmgA* and the 3’ end of BAS0227. BAS0227 is predicted to encode a fumarylacetoacetate hydrolase (HmgB), the final enzyme in a tyrosine and phenylalanine catabolism pathway that converts fumarylacetoacetate to fumarate and acetoacetate in many species.

### Characterization of directed *hmgA* and *hmgB* deletion mutants

**Fig 2:**
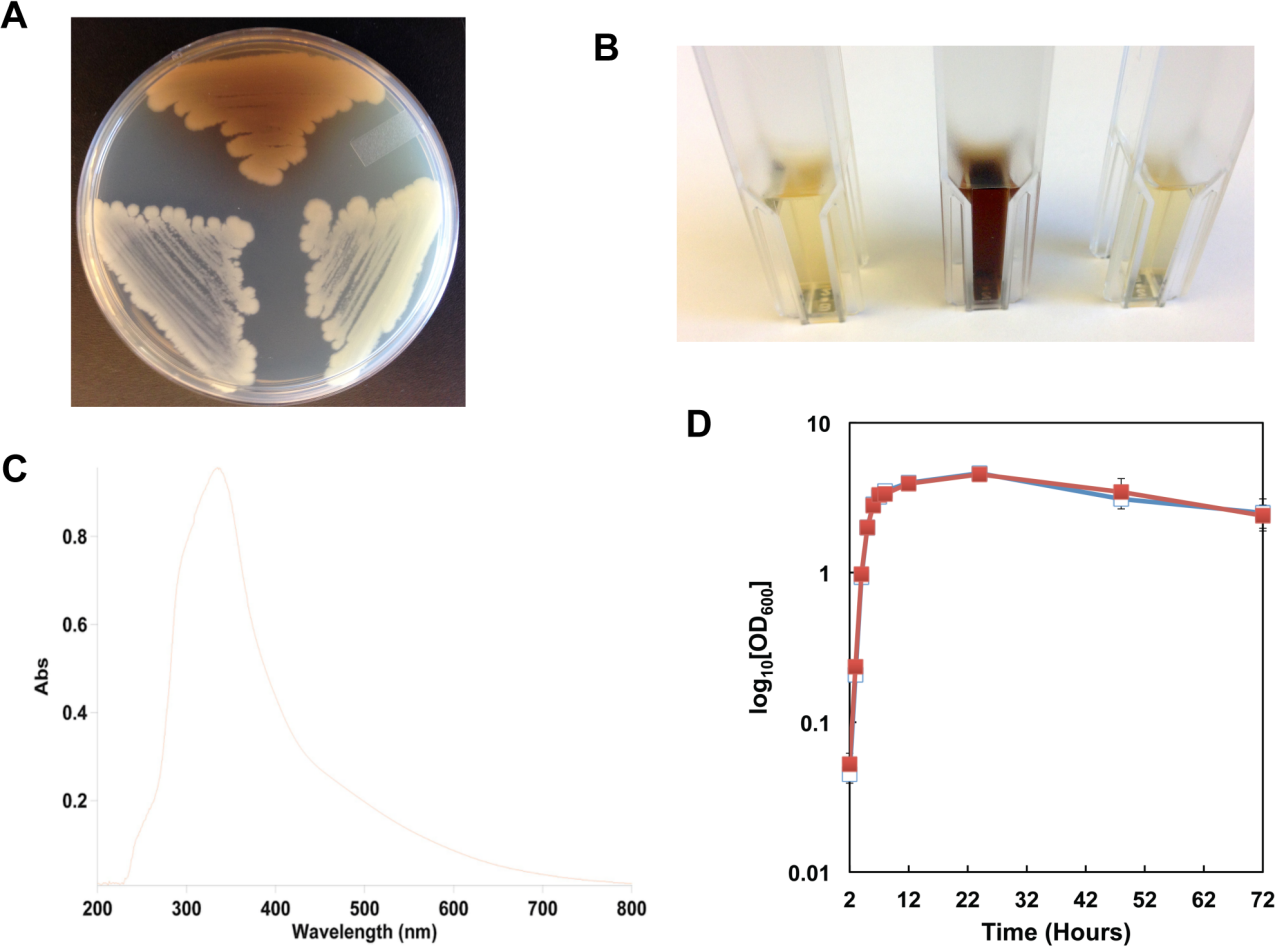
Characteristics of Δ*hmgA* mutant strain. **A.** Pigment production in strains grown on LB-agar for 48 hours at 37°C. Top, AW-A127 (Δ*hmgA*); Bottom left, parental 34F2; Bottom right, AW-A130 (Δ*hmgB*). **B.** Pigment production in supernatants of strains grown in LB for 72 hours at 37°C. Left, 34F2; Center, AW-A127 (Δ*hmgA*); Right, AW-A130 (Δ*hmgB*). **C.** Optical spectra of AW-A127 (Δ*hmgA*) supernatant following subtraction of 34F2 supernatant spectra. **D.** Cell growth of parental and mutant strains grown in LB at 37°C. -■- 34F2; -□- AW-A127 (Δ*hmgA*).

To confirm the phenotype of the transposon mutant, a strain containing a marker-less deletion of *hmgA*, named AW-A127, was created. As with the transposon mutant, directed deletion of *hmgA* resulted in colonies that produce a brown pigment when grown on LB-agar (Fig 2A). When grown in liquid media, brown color is observed in the Δ*hmgA* strain beginning as the cells enter stationary phase, after approximately 5 hours post inoculation, and pigment continues to accumulate in the cultures as incubation continues. Fig 2B demonstrates the extent of pigment accumulation in culture supernatants at 72 hours post inoculation. The brown pigment was analyzed by spectroscopy. Fig 2C shows a spectroscopic scan of cell-free supernatants of the parental strain subtracted from the Δ*hmgA* strain. The Δ*hmgA* supernatants generate a strong absorbance peak near OD_400_ that was not found in the parental strain, consistent with observations of pyomelanin in other species [10,14]. Loss of *hmgA* did not affect growth in LB at 37°C relative to the parental strain (Fig 2D).

**Fig 3:**
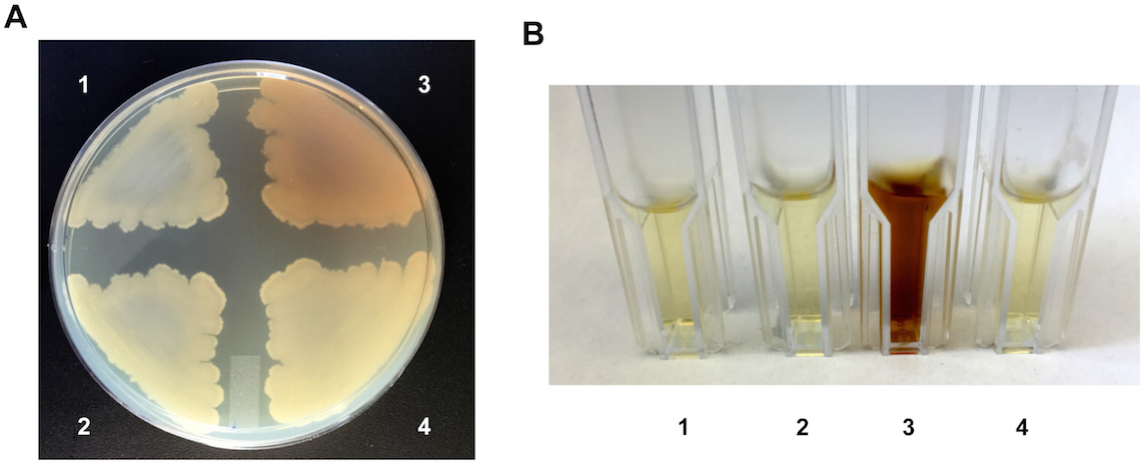
Complementation analysis of Δ*hmgA* mutant strain. **A.** Pigment production in strains grown on LB-agar for 48 hours at 37°C. **B.** Pigment production in supernatants of strains grown in LB for 72 hours at 37°C. For both: 1. 34F2 + pAW285 (empty vector); 2. 34F2 + pAW444 (*hmgA*); 3. AW-A127 (Δ*hmgA*) + pAW285 (empty vector); 4. AW-A127 (Δ*hmgA*) + pAW444 (*hmgA*).

To demonstrate that loss of *hmgA* is responsible for the observed phenotypes, *in trans* complementation analysis was performed. The coding region of *hmgA* was cloned into vector pAW285, producing complementation plasmid pAW444, placing *hmgA* expression under the control of a highly active promoter. The Δ*hmgA* strain carrying the empty plasmid still produces pigment while the Δ*hmgA* strain carrying the *hmgA* complementation plasmid no longer produces pigment (Fig 3). Neither the *hmgA* complementation plasmid nor the empty plasmid affect the parental 34F2 strain.

As the genes predicted to encode *hmgA* and *hmgB* partially overlap, a marker-less deletion of *hmgB*, named AW-A130, was created. As shown in Fig 2A and 2B, loss of *hmgB* does not result in pigment accumulation. Further, loss of *hmgB* does not alter growth characteristics of the strain relative to parental 34F2 (data not shown). The Δ*hmgB* strain was not further analyzed.

### Pyomelanin is produced in presence of L-tyrosine and L-phenylalanine

**Fig 4:**
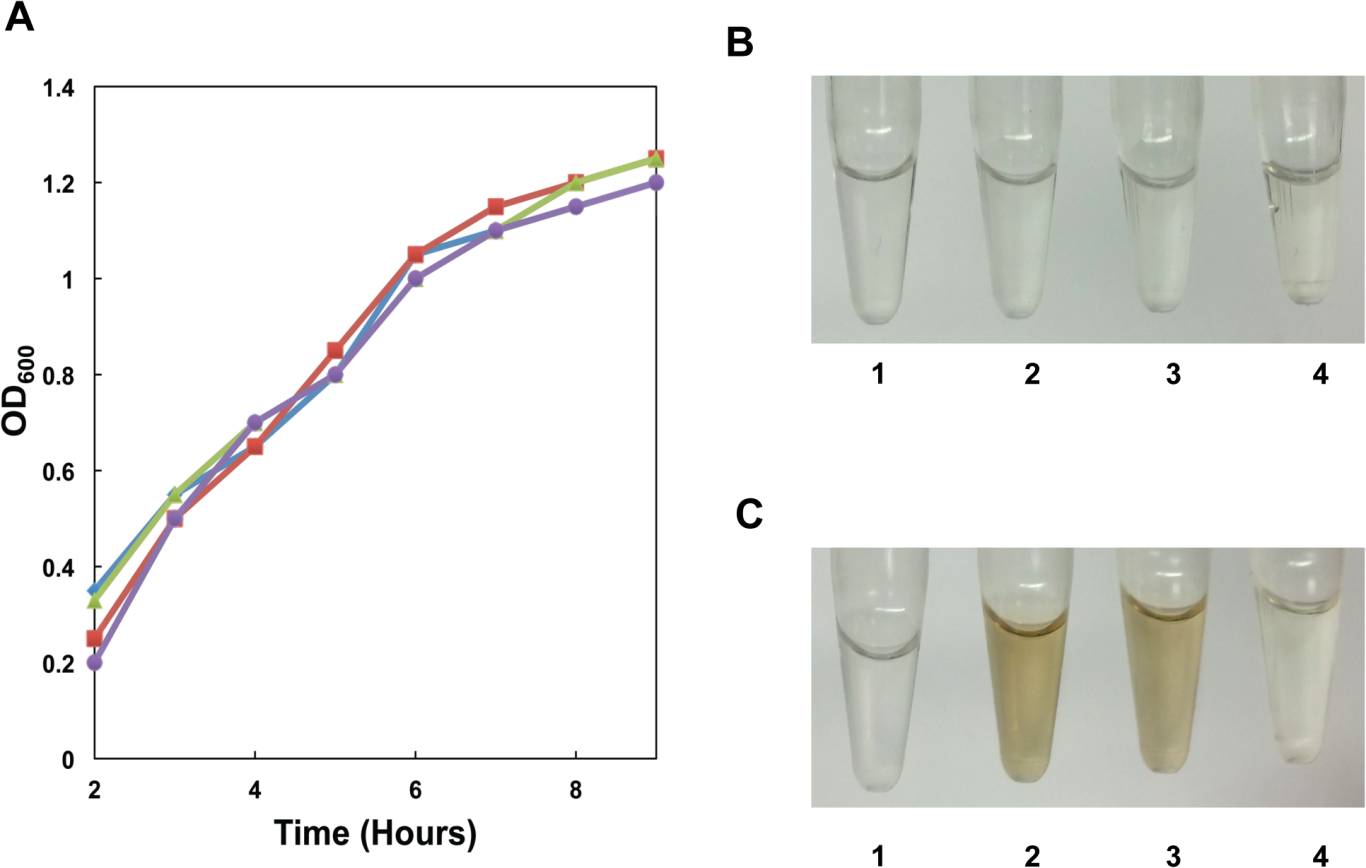
Growth and pigment production in defined medium. **A.** Cell growth of parental and mutant strains grown in R-Bic medium at 37°C. -■- 34F2 in R-bic; -◆- AW-A127 (Δ*hmgA*) in R-bic; -▲- 34F2 in R-bic without L-tryptophan, L-phenylalanine, or L-tyrosine; -●- AW-A127 in R-bic without L-tryptophan, L-phenylalanine, or L-tyrosine. **B.** Pigment production in 34F2 supernatants. **C.** Pigment production in AW-A127 supernatants. 1, R-bic without L-tryptophan, L-phenylalanine, or L-tyrosine; 2, R-bic without L-tryptophan or L-phenylalanine; 3, R-bic without L-tryptophan or L-tyrosine; 4, R-bic without L-phenylalanine or L-tyrosine.

*B*. *anthracis* strains were grown in a derivative of a commonly used defined medium, R-medium, without added bicarbonate (R-bic). Using a defined medium allows removal and addition of specific amino acids to determine contributions to pyomelanin production. Growth of 34F2 and the Δ*hmgA* strain were similar when grown in R-bic or R-bic without L-phenylalanine, L-tyrosine, and L-tryptophan (Fig 4A). Addition or removal of any of these individual amino acids did not alter the growth characteristics of either strain (data not shown). Pigment production did not occur under any conditions tested with parental 34F2 strain (Fig 4B). In the Δ*hmgA* mutant, pigment production occurred in the presence of L-tyrosine and L-phenylalanine but not the presence of L-tryptophan (Fig 4C).

**Fig 5:**
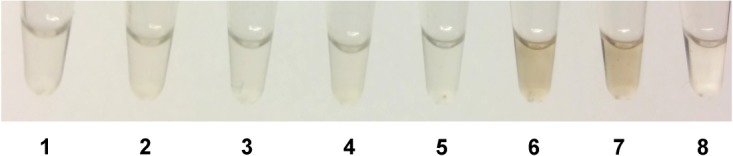
Pigment production in cell-free extracts. Cell-free extracts from cells grown in LB at 37°C for 6 hours. Amino acids then added to cell-free extracts, as indicated, and incubated for an additional 24 hours at 37°C. 1, 34F2, no added amino acids; 2, 34F2 with L-tyrosine; 3, 34F2 with L-phenylalanine; 4, 34F2 with L-tryptophan; 5, AW-A127 (Δ*hmgA*), no added amino acids; 6, AW-A127 with L-tyrosine; 7, AW-A127 with L-phenylalanine; 8, AW-A127 with L-tryptophan.

To confirm these findings, pigment production was tested in cell-free extracts of the parental and mutant strains. 34F2 and the Δ*hmgA* strains were grown to early-stationary phase at 37°C in LB, cells disrupted by sonication, and cell debris removed by filtration. Amino acids were then added to the cell-free extracts and incubated for 48 hours at 37°C. With extracts of the parental strain, pigment was not produced under conditions tested (Fig 5). In extracts of the Δ*hmgA* strain, pigment was observed with the addition of exogenous L-tyrosine or L-phenylalanine but not with the addition of exogenous L-tryptophan. Combined, these findings indicate that, in the Δ*hmgA* strain, pyomelanin is produced in the presence of L-tyrosine and L-phenylalanine.

### Pyomelanin protects *B*. *anthracis* from UV but not oxidative damage

**Fig 6:**
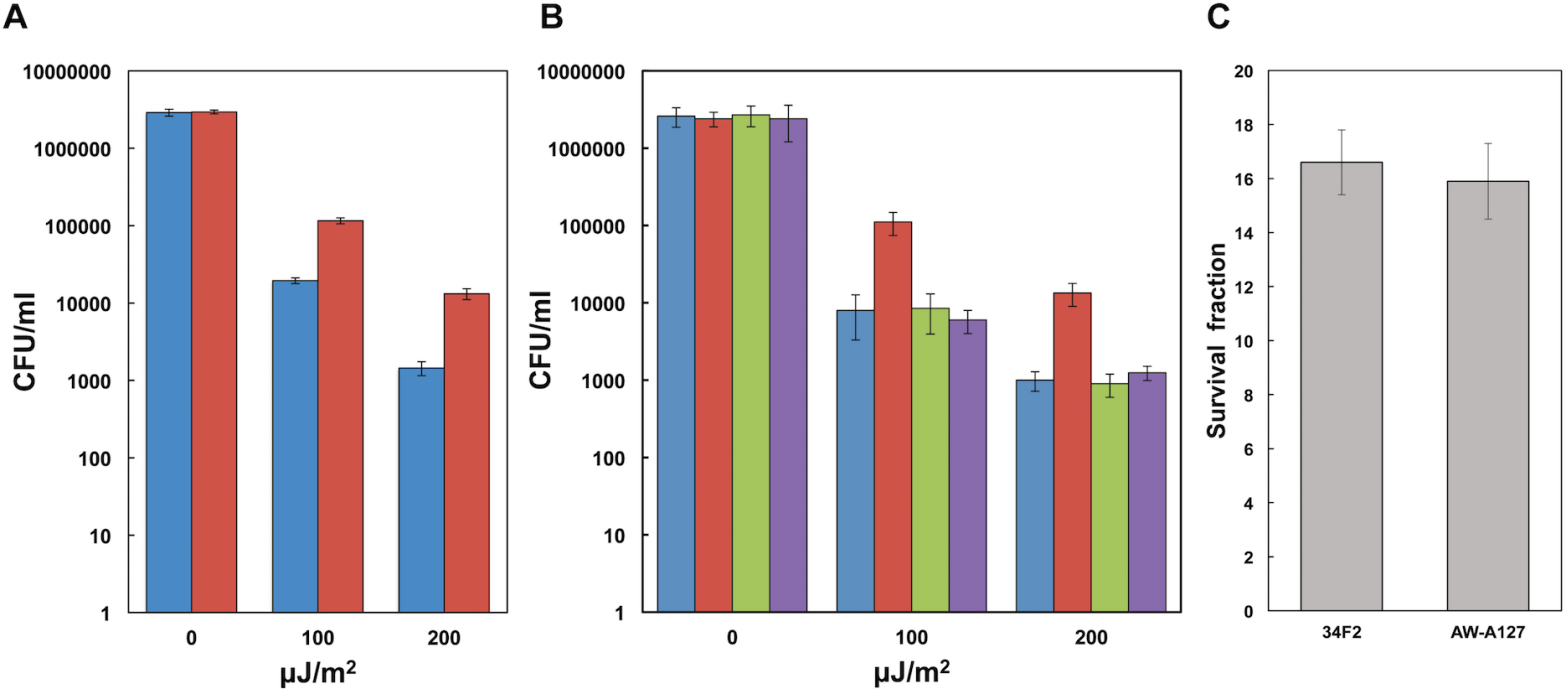
UV and H_2_O_2_ protection. **A.** UV protection. Survival of 34F2 (blue) or AW-A127 (Δ*hmgA*) (red) following irradiation with UV at 302 nm as indicated. Data was obtained from 3 independent cultures and error bars represent standard deviation from the mean. Two tailed unpaired *t* test indicate P value of P<0.0001 between 34F2 and AW-A127 at both levels of UV exposure. **B.** UV protection of complemented strains. Survival of 34F2+pAW285 (blue), AW-A127+pAW285 (red), 34F2+pAW444 (green), and AW-A127+pAW444 (purple) following irradiation with UV at 302 nm as indicated. Data was obtained from 3 independent cultures and error bars represent standard deviation from the mean. **C.** H_2_O_2_ survival. Survival of 34F2 cells in 50mM H_2_O_2_ when resuspended in supernatants from 34F2 or AW-A127 (Δ*hmgA*) cultures. Data was obtained from 3 independent cultures and error bars represent standard deviation from the mean.

Reports from other organisms indicate that production of pyomelanin protects cells from UV and/or H_2_O_2_-mediated oxidative stress [9,10,14,24]. UV sensitivity was tested as previously described [10]. Significantly more cells of the pyomelanin-producing Δ*hmgA* strain survived exposure to UV as compared to the parental 34F2 (Fig 6A). Complementation of *hmgA in trans* increases sensitivity of the Δ*hmgA* strain to UV radiation to parental 34F2 levels (Fig 6B), indicating that pyomelanin production confers protection from UV radiation.

Initially, H_2_O_2_-sensitivity was tested by a simple disk diffusion assay using paper discs saturated with several different concentrations of H_2_O_2_. No difference in the sizes of zones of inhibition were observed between 34F2 and the Δ*hmgA* strain in a range between 1 mM and 200 mM of H_2_O_2_ (data not shown). The protective effects of pyomelanin-containing supernatants on the 34F2 strains were assayed similar previous studies [9,24]. As shown in Fig 6C, no significant difference was seen in the ability of 34F2 or Δ*hmgA* supernatants to protect 34F2 against H_2_O_2_ damage, suggesting that pyomelanin does not protect *B*. *anthracis* against H_2_O_2_-induced oxidative damage.

### Loss of *hmgA* does not alter virulence gene expression

**Fig 7:**
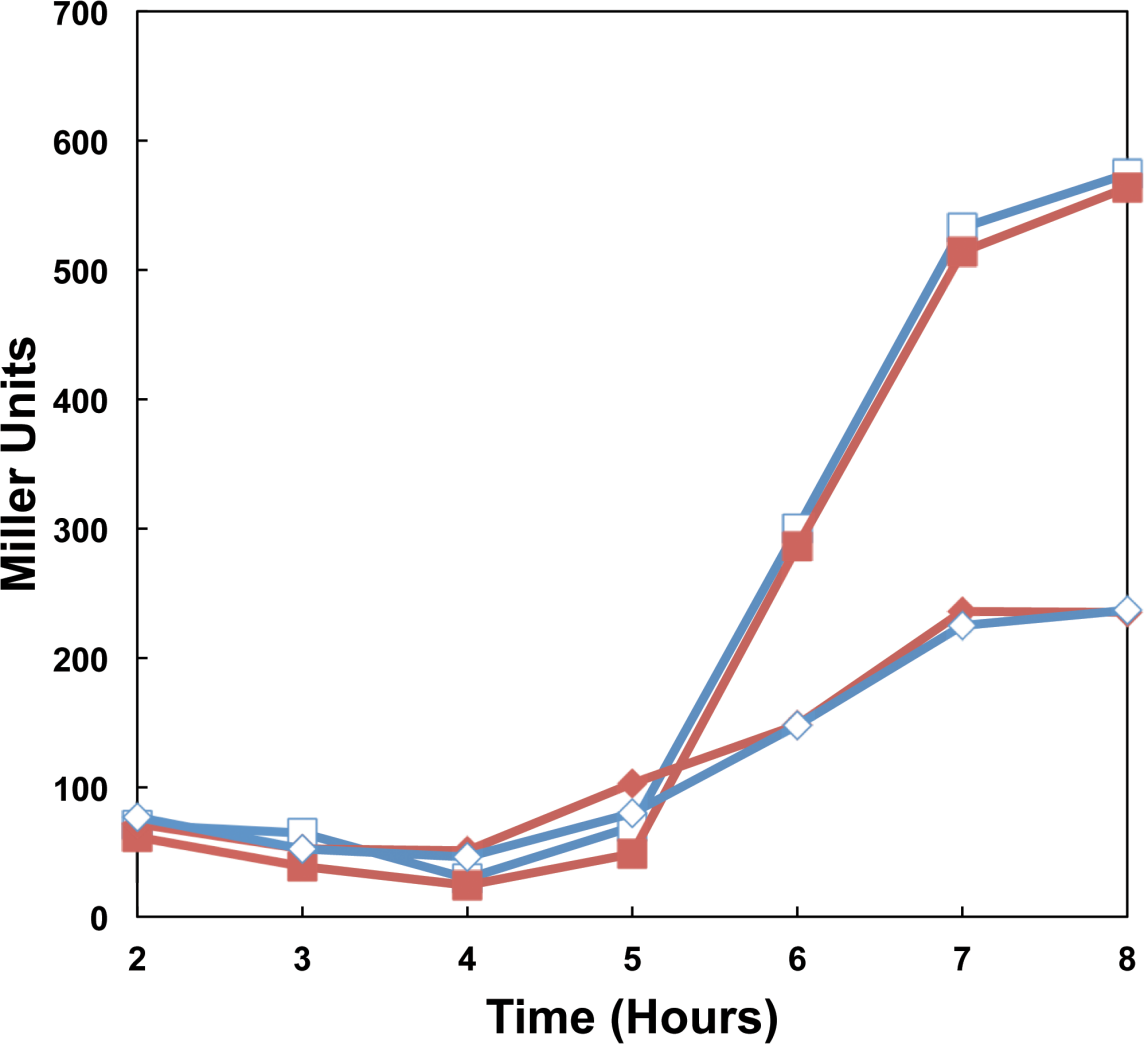
Virulence gene expression in 34F2 and Δ*hmgA* strains. β-galactosidase activity in virulence gene reporter strains grown in LB supplemented with kanamycin at 37°C. -■- 34F2 *pagA*-*lacZ*; -□- AW-A127 *pagA*-*lacZ* -◆- 34F2 *atxA*-*lacZ*; -◇- AW-A127 *atxA*-*lacZ*;.

As production of anthrax toxin is important to virulence of *B*. *anthracis*, the effect of loss of *hmgA* on virulence gene expression was assayed by β-galactosidase analysis. Transcriptional fusions of the promoters of a toxin subunit, *pagA*, and the master virulence regulator, *atxA*, to a *lacZ* reporter were introduced into the 34F2 and Δ*hmgA* strains. No difference in β-galactosidase activity was observed with either reporter between the 34F2 and Δ*hmgA* strains in exponential or stationary phase growth (Fig 7). No difference in β-galactosidase activity was seen in samples allowed to grow to 24 and 48 hours, when pigment accumulation was high (data not shown). These data suggest that neither loss of *hmgA* nor production of pyomelanin significantly affect virulence gene expression.

### Loss of *hmgA* does not alter sporulation or germination

The processes of sporulation and germination are essential to the life cycle of *B*. *anthracis*. As this is the first exploration of HmgA in a sporulating bacterium, the effects of loss of *hmgA* on sporulation and germination were investigated. Sporulation was assayed by a liquid sporulation assay. Sporulation efficiency, as measured by percentage of spores relative to the total number of viable cells from three independent cultures, for the 34F2 strain was 48.9%±7.9% while the Δ*hmgA* strain was 49.3%±1.2%. Germination analysis was performed by calculating CFU from a fixed number of purified spores from three independent spore preparations. For 34F2, 7.7x10^6^±4.1x10^4^ CFU/ml were recovered while for the Δ*hmgA* strain 8.0x10^6^±3.6x10^4^ CFU/ml were recovered. These data suggest that loss of *hmgA* and pigment production affect neither sporulation nor germination in *B*. *anthracis*.

## Discussion

Loss of *hmgA* in *B*. *anthracis* results in accumulation of pyomelanin in the presence of L-tyrosine and L-phenylalanine. Neither the loss of *hmgA* nor the accumulation of pyomelanin affects the growth characteristics of *B*. *anthracis*, indicating that this aromatic amino acid catabolism pathway is not essential. It is unclear if the complete L-tyrosine and L-phenylalanine catabolism pathway with homogentisate as an intermediate is present in *B*. *anthracis*. *B*. *anthracis* carries a putative pterin-dependent phenylalanine hydroxylase (BAS4253) which could function to convert L-phenylalanine to L-tyrosine, allowing L-phenylalanine to be degraded in the homogentisate pathway. A number of genes are predicted to encode aminotransferases (BAS1428, BAS1454, and BAS2746) potentially capable of converting L-tyrosine to 4-hydroxyphenylpyruvate. A predicted 4-hydroxyphenylpyruvate dioxygenase, which converts 4-hydroxyphenylpyruvate to homogentisate, is encoded by BAS0226. HmgA (BAS0228) then converts homogentisate to maleylacetoacetate. The next steps in maleylacetoacetate degradation are unclear. BAS0227 encodes a predicted fumarylacetoacetate hydrolase that converts fumarylacetoacetate to fumarate and acetoacetate, but a gene encoding maleylacetoacetate isomerase, converting maleylacetoacetate to fumarylacetoacetate, cannot be identified in the *B*. *anthracis* genome. A previous report indicates that in some *Bacillus* species, maleylacetoacetate can be degraded by a maleylacetoacetate hydrolase to acetoacetate and maleic acid [29]. A gene encoding a maleylacetoacetate hydrolase also cannot be directly identified in the *B*. *anthracis* genome. Total transcriptome analysis indicates that BAS0226, BAS0227 (*hmgB*), and BAS0228 (*hmgA*) are coordinately transcribed as part of an operon [30], suggesting a connection between the activities of the HmgA and HmgB. The fate of maleylacetoacetate in the homogentisate pathway requires additional analysis.

The production of pigment in cell free extracts of cells in the early stationary phase of growth indicates that the enzymes required to convert L-phenylalanine and L-tyrosine to homogentisic acid are produced at this time; however, pigmentation in batch culture is just becoming detectable in early stationary phase. The appearance of pigment relies on conversion of 4-hydroxyphenylpyruvate to homogentisic acid followed by spontaneous oxidation of homogentisic acid to pyomelanin [11]. When batch cultures of the Δ*hmgA* strain are grown without agitation, the development of pigment is delayed compared to a culture grown with agitation controlled for differences in cell density (data not shown), likely due to decreased oxidation of homogentisic acid. The rate of homogentisic acid production and the oxidation of homogentisic acid to pyomelanin cannot be established from these experiments. Further analysis of homogentisic acid production and oxidation under specific conditions would be needed to optimize pyomelanin production.

*hmgA*-like genes are not found widely in Gram-positive bacteria. In fact, *hmgA*-like genes in the firmicutes are largely restricted to the *Bacillus cereus* group, which includes *B*. *anthracis*, *B*. *cereus*, and *B*. *thuringiensis*. The genome of *Bacillus subtilis*, the model organism for *Bacillus* physiology and genetics, does not encode a predicted homogentisate 1,2-dioxygenase. *Bacillus subtilis* does produce a protein coat that contains a brown pigment around the endospore. This melanin-like pigment is produced by the copper-dependent laccase, CotA [31]. CotA confers resistance to UV light and hydrogen peroxide damage [31,32]. *B*. *anthracis* does possess a CotA-like protein [33], but this protein would produce pigment in the spore, not in vegetative cells as with the loss of HmgA.

The relevance of pyomelanin accumulation to pathogenesis of *B*. *anthracis* is unclear. Neither loss of *hmgA* nor pyomelanin accumulation affect expression of key virulence factors. Pyomelanin production does not appear to protect *B*. *anthracis* cells from oxidative damage, a feature important to anthrax pathogenesis [34]. While pyomelanin protects vegetative cells from UV damage, *B*. *anthracis* would mostly likely be exposed to UV radiation outside the host when in the form of UV-resistant spores. It may be possible that pyomelanin protects cells from other stresses found during the course of infection that were not assayed. Further analysis of pyomelanin-producing mutants in cellular and animal models of infection may be of value.
